# Efficacy and Safety of Tanshinone for Chronic Kidney Disease: A Meta-Analysis

**DOI:** 10.1155/2020/3091814

**Published:** 2020-01-13

**Authors:** Yao Zhou, Shi-min Jiang, Li Li, Ying Wang, Lei Ding, Chao-xia Liu, Qi Wu, Kun Gao

**Affiliations:** ^1^Department of Pathophysiology, Xuzhou Medical University, Xuzhou 221009, China; ^2^Laboratory of Clinical and Experimental Pathology, Xuzhou Medical University, Xuzhou 221009, China; ^3^Department of Physiology, Xuzhou Medical University, Xuzhou 221009, China; ^4^Division of Nephrology, Jiangsu Province Hospital of Chinese Medicine, Nanjing 210029, China; ^5^Affiliated Hospital of Nanjing University of Chinese Medicine, Nanjing 210029, China

## Abstract

**Objective:**

To systematically evaluate the efficacy and safety of tanshinone for chronic kidney disease (CKD).

**Methods:**

Randomized controlled trials (RCTs) on the treatment of CKD using tanshinone were searched using 4 Chinese databases (China National Knowledge Infrastructure (CNKI), Value In Paper (VIP), Wanfang, and Chinese Biology Medicine (CBM)) and 3 English databases (PubMed, Cochrane Library, and Excerpta Medica Database (Embase)). The results included data on blood urine nitrogen (BUN), serum creatinine (Scr), glomerular filtration rate (GFR), 24 h urine protein, microalbuminuria (mALB), *β*2-macroglobulin (*β*2-MG), cystatin C (CysC), and safety events. The data were analyzed using Revman 5.3 and Stata 12.0 software.

**Results:**

Twenty-one studies were entered into this meta-analysis, which involved 1857 patients including 954 cases from the tanshinone treatment group and 903 cases from the control group. BUN levels in the tanshinone treatment group were significantly reduced compared with the control (standardized mean difference (SMD) = −0.65, 95% confidence interval (CI): −0.81 to −0.49, *p* < 0.01). In addition, subgroup analysis indicated that tanshinone had a significant effect in reducing Scr levels at 14, 21, and 28 days. Scr levels in the tanshinone treatment group were significantly reduced compared with the control group (SMD = −1.40, 95% CI: −2.09 to −0.71, *p* < 0.01); subgroup analysis based on treatment time also yielded the same results. GFR in the tanshinone treatment group was better than that in the control group (SMD = 0.83, 95% CI: 0.59 to 1.07, *p* < 0.01). In terms of urine protein levels, 24 h urine protein level, mALB, and *β*2-MG of CKD patients were reduced to some degree compared with controls, and CysC levels in the tanshinone treatment group were also significantly reduced compared with the control group (SMD = −0.24, 95% CI: −0.44 to −0.03, *p* < 0.05). Safety in the tanshinone treatment group did not differ significantly from that of the control group (risk ratio (RR) = 7.78, 95% CI: 0.99 to 61.05, *p* > 0.05).

**Conclusion:**

This meta-analysis showed that tanshinone could control urine protein level in CKD patients, improve kidney function, and delay the evolution of CKD without significant side effects. However, the results were limited and should be interpreted with caution because of the low quality of the included studies. In the future, more rigorous clinical trials need to be conducted to provide sufficient and accurate evidence.

## 1. Introduction

Chronic kidney disease (CKD) is a condition of functional and structural dysfunction of the kidney with impairment longer than 3 months, caused by various factors. It is characterized by normal or abnormal glomerular filtration rate (GFR) from pathological glomerular injury, abnormal renal function indices in urine or blood, abnormal imaging findings, or decreased GFR for more than 3 months of unknown etiology (<60 mL/min·1.73 m^2^) [1]. With increasing life expectancy and parallel increases in the standard of living, the incidence of metabolic diseases such as diabetes, hyperlipidemia, and hypertension is consistently on the rise, leading to annual increases in the incidence of CKD [2].

However, there are presently no effective drugs for treating CKD. CKD is treated mainly with hormone therapy and immune suppressors in addition to control urine protein, blood pressure, and blood glucose levels and maintaining homeostasis. Although these therapies can reduce kidney impairment to a certain degree, they are unable to radically delay the evolution of this disease. Furthermore, hormone and immune suppressors are only effective in part of the pathogenesis of kidney injury and lead to high rates of relapse. Their long-term application can also lead to severe infection and osteonecrosis of the femoral head, among other side effects [3, 4]. Therefore, safe and efficacious drugs are needed for CKD treatment. Tanshinone is the main active ingredient of the frequently used traditional Chinese medicine Danshen. This agent functions to protect body tissues by inducing dilation of the cardiocerebral-renal vasculature, increasing blood flow to ischemic myocardial tissue, scavenging reactive oxygen species (ROS) resulting from lipid peroxidation during organ ischemia/reperfusion injury, and inhibiting intracellular calcium overload. Tanshinone has been widely administered in patients with angina pectoris and myocardial infarction [5, 6]. However, research on CKD revealed that tanshinone can also protect the kidney and delay diabetes-related renal fibrosis [7]. Clinically, a number of studies have shown that tanshinone administration can restore a certain degree of kidney function in CKD patients, but this conclusion has yet to be confirmed.

In the present study, published randomized controlled studies (RCTs) on CKD treatment with tanshinone were found, and the efficacy and safety of tanshinone for CKD were systematically evaluated. The results of this meta-analysis provide some evidence for the clinical application of tanshinone.

## 2. Data and Methods

### 2.1. Literature Search

This search was conducted according to the Preferred Reporting Items for Systematic Reviews and Meta-Analyses (PRISMA) statement for the conduct of meta-analyses of intervention studies [8]. Four Chinese databases (China National Knowledge Infrastructure (CNKI), Value In Paper (VIP), Wanfang, and Chinese Biology Medicine (CBM)) and 3 English databases (PubMed, Cochrane Library, and Excerpta Medica Database (Embase)) were used for the literature search. Conference papers and academic dissertations were searched manually. In cases where the information was incomplete, the corresponding authors of those papers were contacted and the information necessary for the meta-analysis was obtained. The literature search was concluded on June 1, 2019, and therefore included papers published before this date.

The search terms used were (Tanshinone OR Salvia OR Miltiorrhiza OR Danshentong OR Tanshinone IIA OR Tanshinone IIB OR Tanshinone Injection OR Tanshinone Capsule OR Salvia Injection OR Salvia Capsule OR Miltiorrhiza Injection OR Miltiorrhiza Capsule OR Danshentong Injection OR Danshentong Capsule) AND (CKD OR Chronic Kidney Disease OR Kidney Diseases OR Kidney Insufficiency OR Chronic Kidney Failure OR End-stage Kidney Disease OR End-stage Kidney Failure OR Kidney Injury OR Diabetic Kidney Disease OR Hypertensive Kidney Disease OR Kidney Dialysis OR Chronic Renal Disease OR Renal Diseases OR Renal Insufficiency OR Chronic Renal Failure OR End-stage Renal Disease OR End-stage Renal Failure OR Renal Injury OR Diabetic Renal Disease OR Hypertensive Renal Disease OR Renal Dialysis OR Nephrosis OR Diabetic Nephropathy OR Hypertensive Nephropathy OR Nephrotic Syndrome OR Kidney Transplantation OR Cardiorenal Syndrome).

### 2.2. Criteria for Literature Inclusion

#### 2.2.1. Type of Research

RCTs about CKD treatment with tanshinone published domestically and abroad before June 1, 2019, were included.

#### 2.2.2. Study Subjects

The subjects in the RCTs were patients diagnosed with CKD. Diagnostic criteria were based on the relevant guidelines for CKD diagnosis and treatment.

#### 2.2.3. Preventative Measures

The treatment group comprised patients treated with tanshinone, and treatment with other medications plus tanshinone was also included. The control group was treated with placebo or conventional western medicine, and any form of tanshinone treatment was excluded.

#### 2.2.4. Results

At least one of the following results was included: blood urine nitrogen (BUN), serum creatinine (Scr), glomerular filtration rate (GFR), 24 h urine protein, microalbuminuria (mALB), *β*2-macroglobulin (*β*2-MG), cystatin C (CysC), and safety events.

#### 2.2.5. Criteria for Literature Exclusion

The exclusion criteria for literature were as follows: (1) cases not definitively diagnosed with CKD, (2) repeatedly published articles, (3) too little information reported to be useful, and (4) the number of cases and treatments not accurately provided.

### 2.3. Data Extraction

Data were extracted collaboratively by two researchers. They independently extracted data consistent with the study requirements, recorded the data sequentially in a prepared information sheet, and crosschecked the data. Disagreement was resolved by having a third party to make the decision.

### 2.4. Assessment of Literature Quality

The literature included in this meta-analysis was subject to quality assessment according to the Cochrane criteria [9, 10]. The assessed content included random design, blind concealment, blinding, completeness of the final data, selectively reported research results, and publication bias. The risk of each content was evaluated. The risk assessment table was produced using Revman 5.3 software.

### 2.5. Statistical Analysis

Stata 12.0 and Revman 5.3 software were both used to analyze the data. For consecutive variables, the mean value ± standard deviation (SD) and 95% confidence interval (CI) represented the efficacy of treatment. For binary outcome data, risk ratio (RR) was used to calculate the summary. Heterogeneity was tested by the chi-squared test and *I*^2^ test. A random-effects model was used when a chi-squared test (*p* < 0.10) or *I*^2^ > 50% revealed significant evidence of homogeneity across the studies, or else a fixed-effects model was used. Publication bias was evaluated using Begg's test. *p* values less than 0.05 indicated statistical significance. If the number of included trials was sufficient, sensitivity analysis was used to assess the stability of the results.

## 3. Results

### 3.1. Results of Our Literature Search

**Figure 1:**
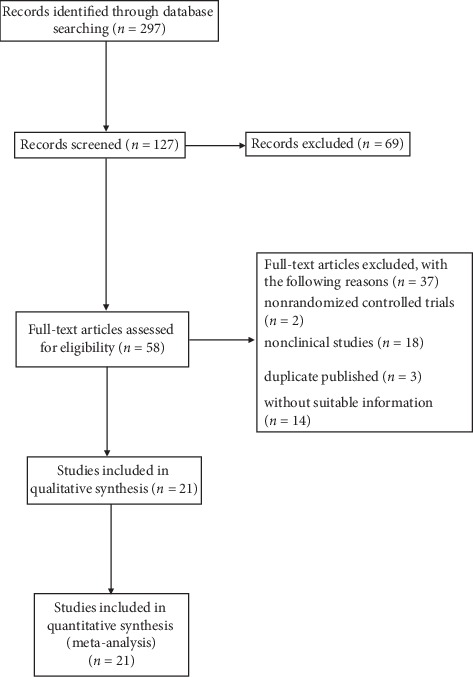
Flowchart showing the process of study selection.

Computer searches and manual searching resulted in 297 publications, of which 127 potentially eligible reports were identified. First, 69 articles were excluded based on identification of titles and abstracts. Next, 2 single-arm studies, 18 animal studies, 3 repeated publications, and 14 articles with uncertain diagnosis or preventative measures were excluded after reading the entire article. Finally, 21 studies were included in the meta-analysis (Figure 1).

### 3.2. Basic Characteristics of the Included Studies

In total, 21 studies were included in the present meta-analysis, which involved 1875 patients comprising 954 patients from the tanshinone treatment group and 903 patients from the control group. Authors, year of publication, treatment plan, sample size, course of treatment, and index of evaluation of each study are shown in Table 1.

### 3.3. Risk of Bias Assessment of the Literature Included in the Study

**Figure 2:**
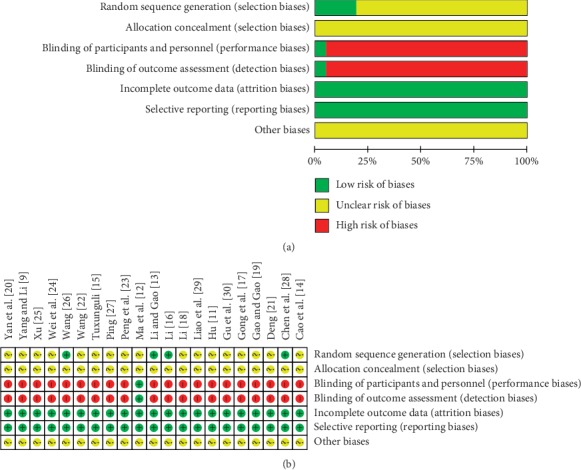
Bias risk analysis of the included studies. (a) Methodological quality assessment of all included studies. (b) Summary of methodological quality assessment of each included study. +: L (low risk of bias); ?: U (unclear risk of bias); −: H (high risk of bias).

The 21 studies included in this analysis were all RCTs, but only 3 had detailed descriptions of the methods. One study had a double-blind design. None of the 21 studies had allocation concealment. We could not confirm any other forms of bias because the available information was insufficient. Assessment of bias risk of the studies in the meta-analysis is shown in Figures 2(a) and 2(b).

### 3.4. BUN

**Figure 3:**
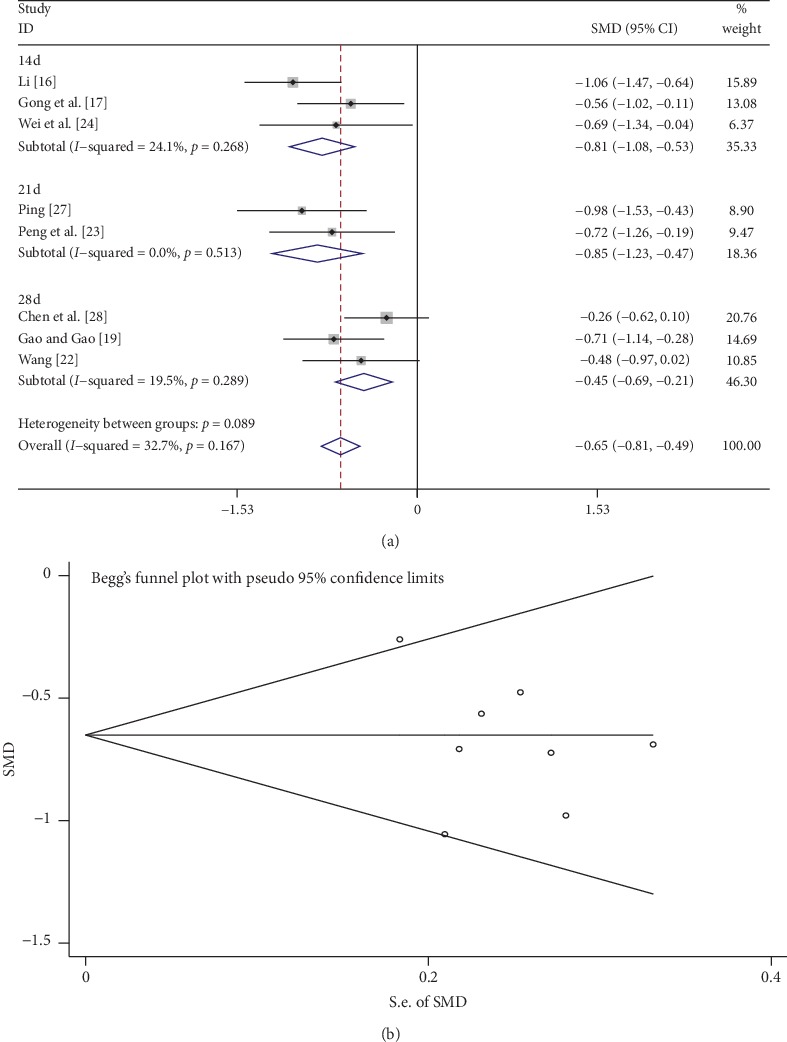
Comparison of BUN levels between the tanshinone treatment and control groups. (a) Forest plots comparing BUN levels between the groups. (b) Funnel plot showing publication bias of BUN between the groups using Begg's rank correlation test.

A total of 8 studies compared BUN levels between the tanshinone treatment group and control group. We classified these studies into different subgroups based on the time of tanshinone treatment, namely, 14, 21, and 28 days. As shown in Figure 3(a), there was low heterogeneity (*I*^2^ = 32.7%, *p* > 0.1), so a fixed-effects model was used to analyze the data. BUN levels in the tanshinone treatment group were significantly reduced compared with the control group (SMD = −0.65, 95% CI: −0.81 to −0.49, *p* < 0.01). The results of subgroup analysis indicated that tanshinone had a significant effect in reducing BUN levels at 14, 21, and 28 days. As shown in Figure 3(b), Begg's test showed that there was no publication bias (*p* > 0.05).

### 3.5. Scr

**Figure 4:**
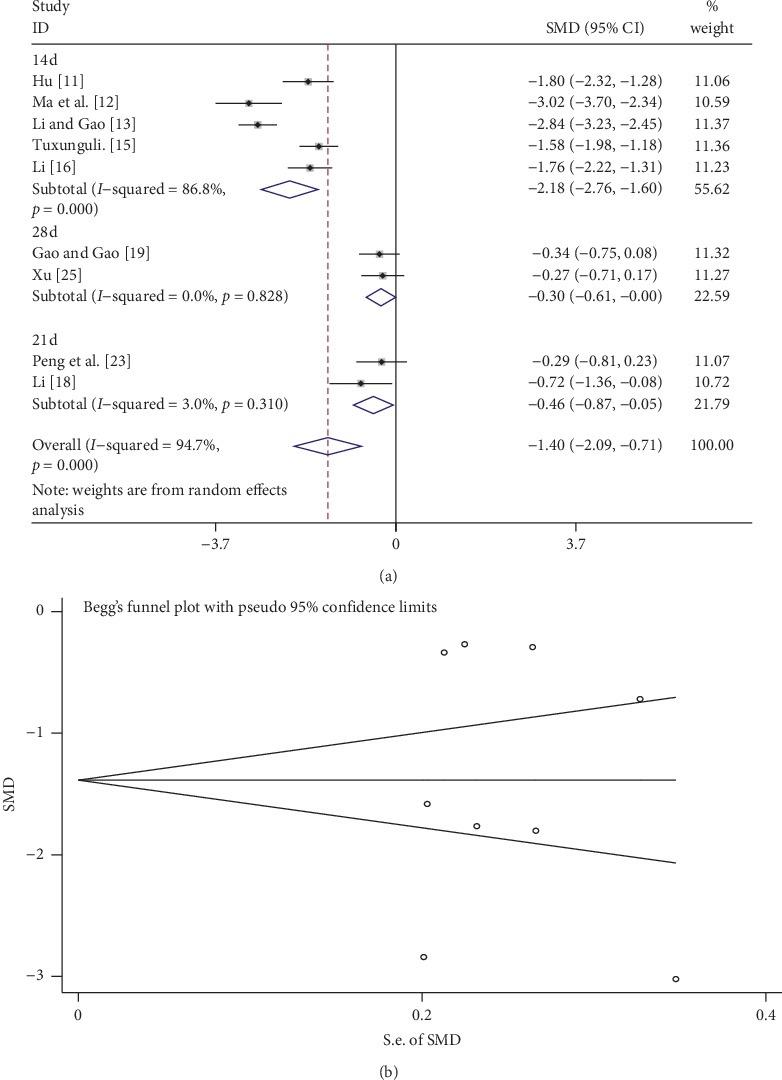
Comparison of Scr in the tanshinone treatment group and control group. (a) Forest plots for comparisons of Scr between the groups. (b) Funnel plot showing Scr publication bias between the groups using Begg's rank correlation test.

A total of 9 studies compared Scr levels between the tanshinone treatment group and control group. We classified these studies into different subgroups based on time of tanshinone treatment, namely, 14, 21, and 28 days. As shown in Figure 4(a), the random-effects model was used to analyze the data because of the high heterogeneity. Scr levels in the tanshinone treatment group were significantly reduced compared with the control group (SMD = −1.40, 95% CI: −2.09 to −0.71, *p* < 0.01). Subgroup analysis indicated that tanshinone had a significant effect on reducing Scr levels for 14, 21, and 28 days. As shown in Figure 4(a), Begg's test showed that there was no publication bias (*p* > 0.05).

### 3.6. GFR

**Figure 5:**
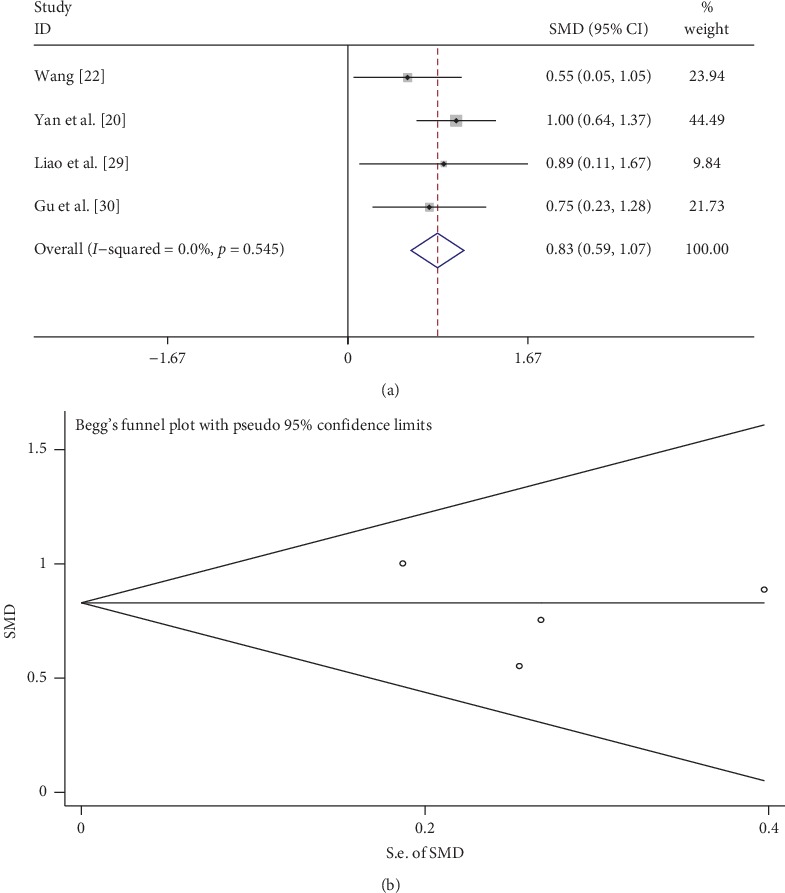
Comparison of GFR between the tanshinone treatment and control groups. (a) Forest plots for comparisons of GFR between the groups. (b) Funnel plot for GFR publication bias between the groups.

A total of 4 studies compared GFR between the tanshinone treatment and control groups. As shown in Figure 5(a), there was low heterogeneity (*I*^2^ = 0%, *p* > 0.1), so the fixed-effects model was used to analyze the data. The tanshinone treatment group had better GFR than the control group (SMD = 0.83, 95% CI: 0.59 to 1.07, *p* < 0.01). Begg's test showed that there was no publication bias (*p* > 0.05; Figure 5(b)).

### 3.7. Quantification of 24 h Urine Protein

**Figure 6:**
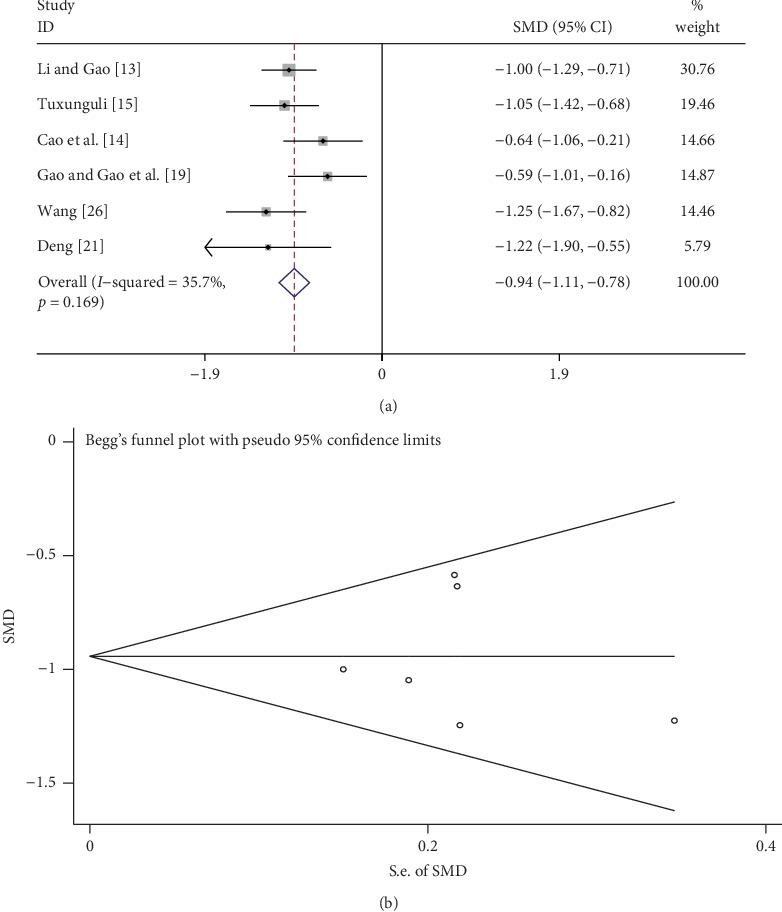
Comparison of 24 h urine protein in the tanshinone treatment and control groups. (a) Forest plots for comparisons of 24 h urine protein between the groups. (b) Funnel plot for 24 h urine protein publication bias between the groups.

A total of 6 studies quantitatively compared the 24 h urine protein between the tanshinone treatment group and control group. As shown in Figure 6(a), the fixed-effects model was used to analyze the data because of low heterogeneity (*I*^2^ = 35.7%, *p* *>* 0.1). Tanshinone significantly reduced 24 h urine protein level compared with the control group (SMD = −0.94, 95% CI: −1.11 to −0.78, *p* < 0.01). Begg's test indicated that there was no publication bias (*p* > 0.05; Figure 6(b)).

### 3.8. mALB

**Figure 7:**
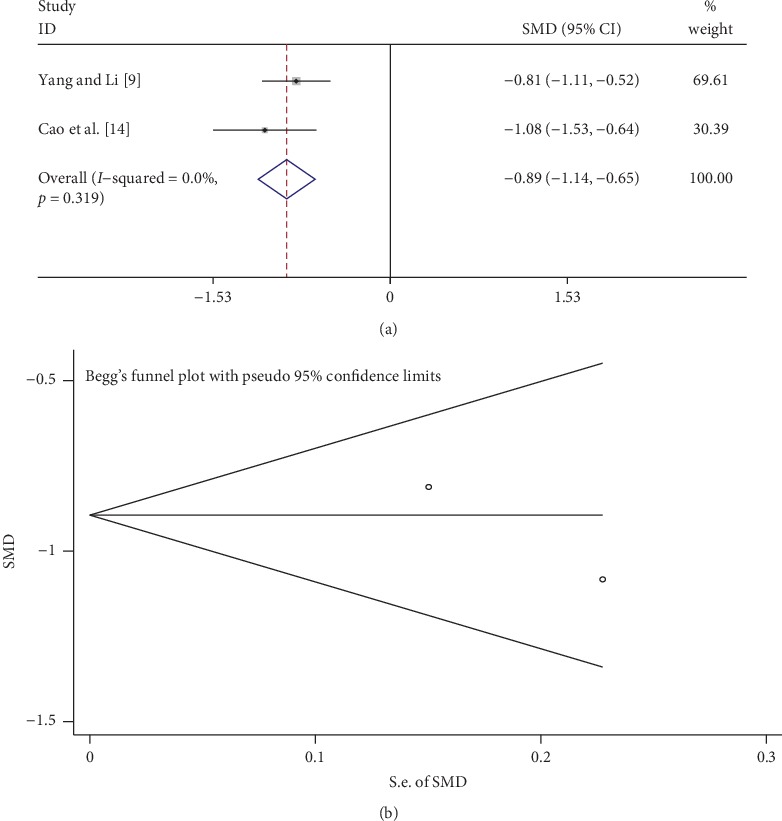
Comparison of mALB in the tanshinone treatment group and control group. (a) Forest plots for comparisons of mALB between the groups. (b) Funnel plot showing mALB publication bias between the groups.

Two studies compared mALB between the tanshinone treatment group and control group. As shown in Figure 7(a), the fixed-effects model was used to analyze the data because of low heterogeneity (*I*^2^ = 0%, *p* > 0.1). Tanshinone significantly reduced mALB levels compared with the control group (SMD = −0.89, 95% CI: −1.14 to –0.65, *p* < 0.01). Begg's test suggested that there was no publication bias (*p* > 0.05; Figure 7(b)).

### 3.9. *β*2-MG

**Figure 8:**
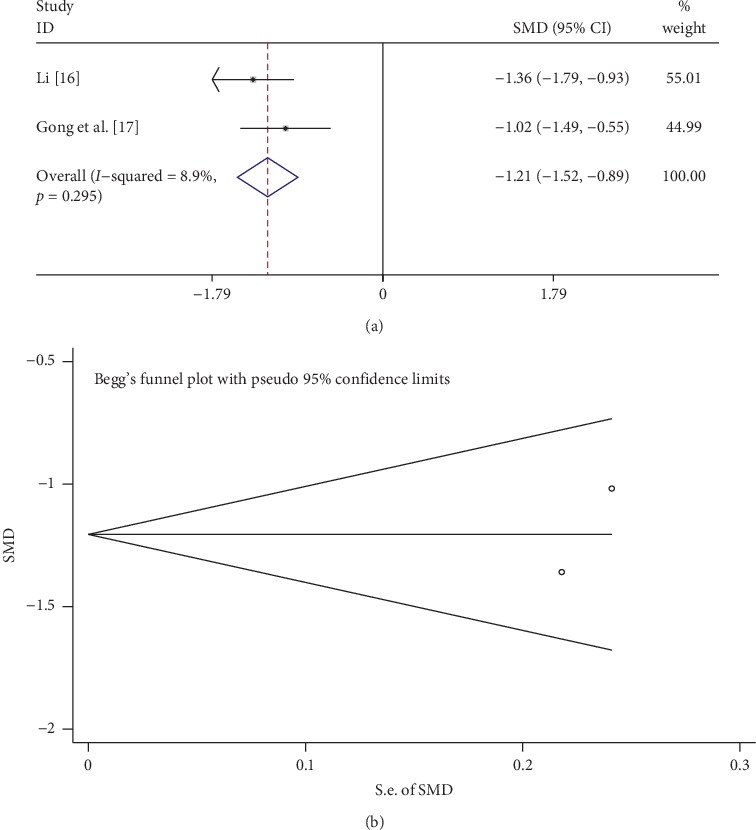
Comparison of *β*2-MG levels in the tanshinone treatment group and control group. (a) Forest plots for comparisons of *β*2-MG between the groups. (b) Funnel plots for *β*2-MG publication bias between the groups.

A total of 3 studies compared *β*2-MG between the tanshinone treatment and control groups. As shown in Figure 8(a), the fixed-effects model was used to analyze the data because of low heterogeneity (*I*^2^ = 8.9%, *p* > 0.1). Tanshinone significantly reduced *β*2-MG levels compared with the control group (SMD = −1.21, 95% CI: −1.52 to −0.89, *p* < 0.01). Begg's test also suggested that there was no publication bias (*p* > 0.05; Figure 8(b)).

### 3.10. CysC

**Figure 9:**
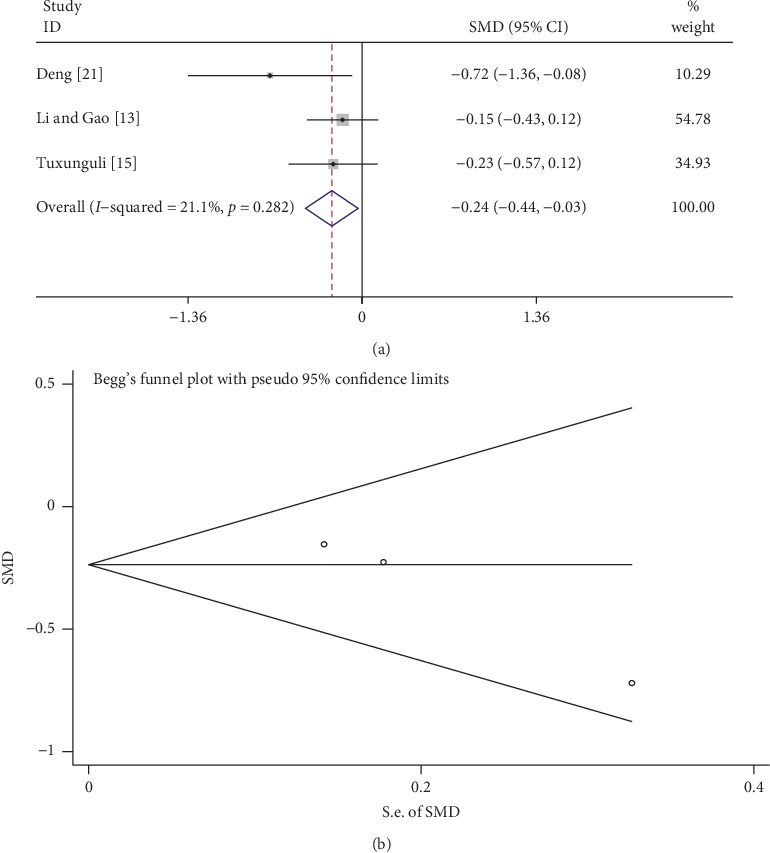
Comparison of CysC levels in the tanshinone treatment and control groups. (a) Forest plots for comparisons of CysC between the groups. (b) Funnel plot for CysC publication bias between the groups using Begg's rank correlation test.

A total of 3 studies compared CysC levels between the tanshinone treatment and control groups. As shown in Figure 8(a), the fixed-effects model was used to analyze the data because of low heterogeneity (*I*^2^ = 21.1%, *p* > 0.1). CysC content in the tanshinone treatment group was significantly lower than that of the control group (SMD = −0.24, 95% CI: −0.44 to –0.03, *p* < 0.05). No publication bias was observed according to Begg's test (*p* > 0.05; Figure 9(b)).

### 3.11. Safety

Two publications addressed the safety of tanshinone in treating CKD.

**Figure 10:**
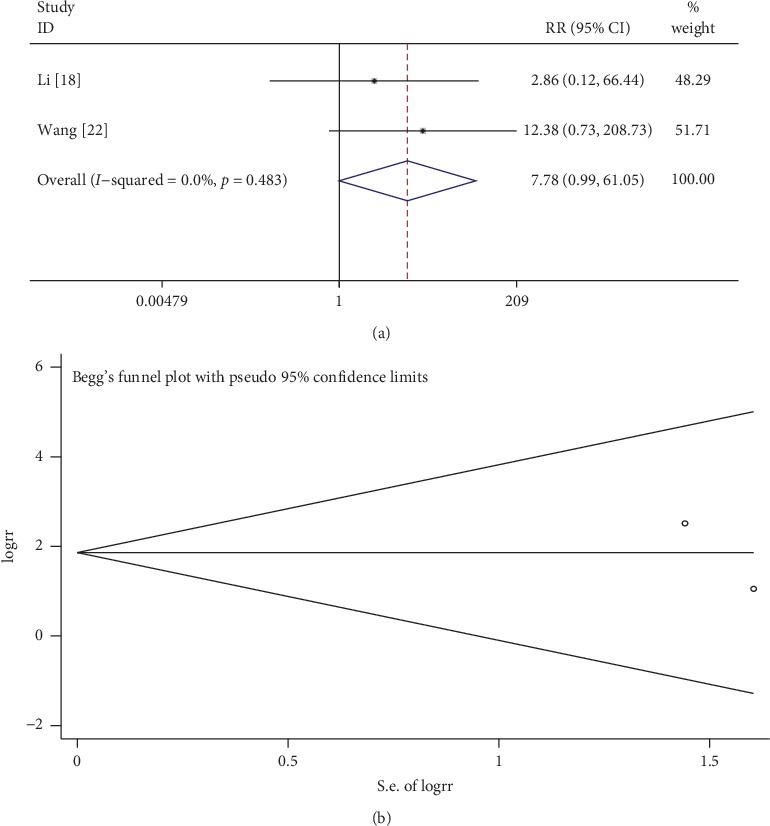
Comparison of safety between the tanshinone treatment and control groups. (a) Forest plots for comparisons of safety between the groups. (b) Funnel plot for the publication bias of safety between the groups using Begg's rank correlation test.

In terms of side effects, 1 article reported mild stomach upset in some patients, and in another study patients complained of slight dizziness, with facial flushing that did not affect treatment in another article. As shown in Figure 10(a), the fixed-effects model was used to analyze the data because of low heterogeneity (*I*^2^ = 0%, *p* > 0.1). Safety in the tanshinone treatment group did not differ significantly from that of the control group (RR = 7.78, 95% CI: 0.99 to 61.05, *p* > 0.05). This suggested that no significant side effects occurred after the addition of tanshinone. Likewise, no publication bias was observed according to Begg's test (*p* > 0.05; Figure 9(b)).

## 4. Discussion

Danshen is a perennial herbaceous plant belonging to the mint family Lamiaceae [31]. Its root is used as a traditional Chinese medicine that has been widely used clinically for the treatment of cardiovascular diseases. Tanshinone is the active ingredient of Danshen and contains more than 50 compounds such as liposoluble compound tanshinone I, tanshinone IIA, and tanshinone IIB as well as the water-soluble compounds salvianolic acid A, salvianolic acid B, and tanshinol [32], of which tanshinone IIB is the most active ingredient, and tanshinone IIA-sodium sulfonate injection is a water-soluble compound derived from the sulfonation of tanshinone, a diterpene quinone compound isolated from Danshen. Many studies have demonstrated its anti-inflammatory, antioxidative, and ROS scavenging activities [33, 34].

CKD is a clinically prevalent, chronic, noninfectious, progressive, and irreversible disease characterized mainly by decreasing GFR. Epidemiological evidence suggests that CKD is mainly the result of glomerular disease and vascular disease caused by diabetes among other reasons [35].

In the present meta-analysis, the effects of tanshinone on CKD were compared with those of the control groups in terms of the three indicators, BUN, Scr, and GFR. Taken together, the results showed that BUN levels significantly decreased in the tanshinone treatment group compared with the control group, and subgroup analysis based on treatment time yielded the same results. For all the CKD patients included in this meta-analysis, Scr levels decreased significantly, while GFR markedly increased. These results suggest that tanshinone protects the kidney in CKD patients and improves GFR.

A common clinical feature of CKD patients is persistent proteinuria. We first compared 24 h urine protein in the tanshinone treatment and control groups. The comparison revealed that tanshinone treatment significantly reduced urine protein, while Begg's test showed that there was no publication bias, indicating that tanshinone effectively reduced urine protein level in CKD patients. To further confirm this, we reviewed literature on mALB, and the combined results showed that tanshinone effectively reduced the mALB levels in CKD patients. Next, we selected 2 publications on the effects of tanshinone treatment on *β*2-MG levels in CKD patients, and the combined data showed that tanshinone treatment significantly reduced *β*2-MG levels in CKD patients. These results indicate that tanshinone has a role in controlling proteinuria in patients with CKD.

Presently, CysC is considered an endogenous marker of GFR, reflecting to a certain extent the filtration capacity of the glomerulus [36]. CysC could be an index of renal function. Three studies on CysC were included in the present meta-analysis. The results showed that tanshinone significantly reduced CysC levels in CKD patients compared with those of the control group. Meanwhile, the results of Begg's test did not indicate publication bias, so the results can be taken as credible. The results of the aforementioned studies suggest that tanshinone could effectively reduce CysC levels in CKD patients and thus protect the kidney.

Only 2 studies evaluated the safety of tanshinone. The combined results showed that there was no significant increase in the side effects seen after the addition of tanshinone, suggesting that tanshinone treatment is relatively safe and has few side effects.

Although the effects of tanshinone on CKD were evaluated using a multiple dimensional meta-analysis, there were some limitations. The quality of the literature included in this present meta-analysis was relatively low, which may have affected the reliability of the results. Although the included literature claimed to have a randomized controlled design, only 3 studies clearly explained their methods, and 1 study used the double-blinded method. When combining the Scr data, the subgroup was highly heterogeneous, which may have impacted the reliability of the results. Limited by language barriers, only Chinese and English databases were searched, and only Chinese studies were included in the meta-analysis, which may have affected the final results to a certain degree.

## 5. Conclusion

Tanshinone controls urine protein level in CKD patients, improves kidney function, and delays the progression of CKD. Meanwhile, no major adverse effects have been reported. However, this conclusion should be interpreted with caution because of the poor quality of the included trials. More rigorous clinical trials including double-blind, randomized, placebo-controlled designs are required to provide more accurate evidence for the future.

## Figures and Tables

**Table 1:** Characteristics of included articles.

Study	Year	Treatment	Control	Number (T/C)	Duration	Results report
Yang and Li [11]	2016	Sulfotanshinone IIA (40–80 mg/d, ivgtt, qd) + western standard treatment	Western standard treatment	96/96	21 d	mALB
Hu [12]	2017	Sulfotanshinone IIA (40 mg/d, ivgtt, qd) + valsartan (80 mg/d, po, qd)	Valsartan (80 mg/d, po, qd)	40/40	14 d	Scr
Ma et al. [9]	2017	Sulfotanshinone IIA (60 mg/d, ivgtt, qd) + valsartan (80 mg/d, po, qd)	Valsartan (80 mg/d, po, qd)	36/36	14 d	Scr
Li and Guo [13]	2017	Sulfotanshinone (60 mg/d, ivgtt, qd) + valsartan (200 mg/d, po, qd)	Valsartan (200 mg/d, po, qd)	100/100	14 d	Scr, CysC, 24-hour proteinuria
Cao et al. [14]	2014	Sulfotanshinone IIA (60 mg/d, ivgtt, qd) + irbesartan (150 mg/d, po, qd)	Irbesartan (150 mg/d, po, qd)	45/44	28 d	mALB, 24-hour proteinuria
Tuxunguli [15]	2017	Sulfotanshinone IIA (60 mg/d, ivgtt, qd) + valsartan (80 mg/d, po, qd)	Valsartan (80 mg/d, po, qd)	64/64	14 d	Scr, CysC, 24-hour proteinuria
Li [16]	2018	Sulfotanshinone IIA (60 mg/d, ivgtt, qd) + alprostadil (10 *μ*g/d, iv, qd)	Alprostadil (10 *μ*g/d, iv, qd)	52/52	14 d	BUN, scr, *β*2-MG
Gong et al. [17]	2012	Sulfotanshinone IIA (40 mg/d, ivgtt, qd) + alprostadil (10 *μ*g/d, iv, qd)	Alprostadil (10 *μ*g/d, iv, qd)	39/39	14 d	BUN, *β*2-MG
Li [18]	2007	Sulfotanshinone IIA (60 mg/d, ivgtt, qd) + western standard treatment	Western standard treatment	20/20	21 d	Scr, safety
Gao and Gao [19]	2011	Sulfotanshinone IIA (50 mg/d, ivgtt, qd) + western standard treatment	Western standard treatment	48/42	28 d	BUN, Scr,24-hour proteinuria
Yan et al. [20]	2011	Sulfotanshinone IIA (50 mg/d, ivgtt, qd) + western standard treatment	Western standard treatment	71/59	15 d	GFR
Deng [21]	2011	Sulfotanshinone IIA (60 mg/d, ivgtt, qd) + western standard treatment	Western standard treatment	20/20	20 d	CysC, 24-hour proteinuria
Wang [22]	2014	Sulfotanshinone IIA (40 mg/d, ivgtt, qd) + + Alprostadil (10 *μ*g/d, ivgtt, qd) + western standard treatment	Western standard treatment	32/32	28 d	BUN, GFR, safety
Peng et al. [23]	2010	Sulfotanshinone IIA (40 mg/d, ivgtt, qd) + haikun shenxi capsule (6 capsules/d, po, tid)	Western standard treatment	30/28	21 d	BUN, scr
Wei et al. [24]	2016	Sulfotanshinone IIA (40 mg/d, ivgtt, qd) + alprostadil (10 *μ*g/d, ivgtt, qd)	Alprostadil (10 *μ*g/d, ivgtt, qd)	27/15	14 d	BUN
Xu [25]	2013	Sulfotanshinone IIA (50 mg/d, ivgtt, qd) + alprostadil (10 *μ*g/d, ivgtt, qd)	Alprostadil (10 *μ*g/d, ivgtt, qd)	40/40	28 d	Scr
Wang [26]	2018	Sulfotanshinone IIA (50 mg/d, ivgtt, qd) + shen kang injection (100 ml/d, ivgtt, qd)	Western standard treatment	50/50	30 d	24-hour proteinuria
Ping [27]	2007	Sulfotanshinone IIA (50 mg/d, ivgtt, qd) + prednisone (1 mg/kg/d, po, qd)	Prednisone (1 mg/kg/d, po, qd)	40/22	21 d	BUN
Chen et al. [28]	2009	Sulfotanshinone IIA (100 mg/d, ivgtt, qd) + prednisone (1 mg/kg/d, po, qd)	Prednisone (1 mg/kg/d, po, qd)	60/60	60 d	BUN
Liao et al. [29]	2009	Sulfotanshinone IIA (0.5 mg/kg/d, ivgtt, qd) + western standard treatment	Western standard treatment	14/14	7 d	GFR
Gu et al. [30]	2013	Sulfotanshinone IIA (0.5 mg/kg/d, ivgtt, qd) + western standard treatment	Western standard treatment	30/30	14 d	GFR

## Data Availability

The data used to support the findings of this study are available from the corresponding author upon request.
